# Bright lights, big city: Causal effects of population and GDP on urban brightness

**DOI:** 10.1371/journal.pone.0199545

**Published:** 2018-07-11

**Authors:** Yuhao Lu, Nicholas C. Coops

**Affiliations:** Integrated Remote Sensing Studio, Forest Recourses Management, University of British Columbia, Vancouver, BC, Canada; Beijing University of Posts and Telecommunications, CHINA

## Abstract

Cities are arguably both the cause, and answer, to societies’ current sustainability issues. Urbanization is the interplay between a city’s physical growth and its socio-economic development, both of which consume a substantial amount of energy and resources. Knowledge of the underlying driver(s) of urban expansion facilitates not only academic research but, more importantly, bridges the gap between science, policy drafting, and practical urban management. An increasing number of researchers are recognizing the benefits of innovative remotely sensed datasets, such as nighttime lights data (NTL), as a proxy to map urbanization and subsequently examine the driving socio-economic variables in cities. We further these approaches, by taking a trans-pacific view, and examine how an array of socio-economic ind0icators of 25 culturally and economically important urban hubs relate to long term patterns in NTL for the past 21 years. We undertake a classic econometric approach—panel causality tests which allow analysis of the causal relationships between NTL and socio-economic development across the region. The panel causality test results show a contrasting effect of population and gross domestic product (GDP) on NTL in fast, and slowly, changing cities. Information derived from this study quantitatively chronicles urban activities in the pan-Pacific region and potentially offers data for studies that spatially track local progress of sustainable urban development goals.

## Introduction

Cities have multi-faceted descriptions, including the permanent areas of heavily human-induced infrastructure and the socio-economic entities that facilitate industrial development and population growth [1,2]. City growth, commonly known as urbanization, is thus the interplay between a city’s physical and socio-economic environment. Reliable assessment and quantification of urbanization is critical to better allocate resources and optimize the efficiency at which cities develop According to recent estimates [3], approximately over 50% of the global human population resides in cities. To accommodate such enormous populations, cities are responsible for nearly 78% of global carbon emissions, 60% of water use, and 76% of wood consumption [4]. Equally substantial is the regional variability across cities. Urbanization has shifted from developed countries to less developed regions such as south-east Asia, which is seeing the fastest and most intense development activities, a trend that is predicted to continue well into this century. In North America, more than 80% of the population lives in urbanized areas, while in Asia this number falls to 48% [3]. Yet from 2000 to 2010 approximately 200 million people migrated from rural environments to cities in East Asia alone [5].

Key reliable, and consistent, measurements of urbanization are critical to understanding this ongoing movement of people into urban environments, and typically fall into two main categories. First consists of demographic metrics such as births, deaths, immigration, and migration, as well as derived estimates of population size and density. The second is associated with the wealth of a city such as regional gross domestic product (GDP). Data can be acquired in a number of ways. Population data are often recorded through census where the resident population is polled locally using forms and interviews. Alternatively, economic data are most often complied directly by state government or local administrative units. Census and economic data are often in tabular format with limited value for monitoring spatially explicit changes that are needed in urban studies [6]. It was not until the 1970s that remote sensing satellite imagery became an alternative data source for monitoring city growth in a more repeatable and comprehensive manner, and has the potential to offer a richer source of information than conventional survey data alone [7]. However, most urban remote sensing applications mainly focused on extracting physical features such as delineating city boundaries [8] or mapping and quantifying land cover changes [9]. Characterizing the socioeconomic nature of cities has still primarily remained the domain of census data.

In 1992 the first digital nighttime lights (NTL) data were acquired by the Defense Meteorological Satellite Program's Operational Linescan System (DMDP/OLS) and was released by NOAA’s National Geographical Data Center (NGDC). NTL has been used extensively to track urban activity and its associated temporal characteristics, enabling researchers and urban planners to quantitatively compare and contrast spatio-temporal patterns. The full NTL temporal record enables us to chronicle the development of urban patterns and produce spatially explicit estimates that reflect a city’s growth or decline [10]. Early studies such as Welch et al. (1980) [11] use NTL to model urban population and energy consumption, while Croft (1973) [12] uses the nighttime space photographs to map burning waste in oil fields [12]. More recently, digital NTL data have been increasingly used on mapping urban and urbanization related human activities such as delineating urban expansion [13], modelling economic activities [14], and CO_2_ emissions [15]. Yet, the interpretation of NTL brightness, also known as Digital Number (DN) values, can be highly subjective and varies from study to study [16]. In this paper we interpret NTL values in a more general fashion to represent overall human activities. Thus, we assume that an increasing NTL value is indicative of increasing human activity rather than directly linked to one specific variable as in previous studies.

Much of the existing research has shown encouraging results correlating NTL with other ancillary variables such as GDP and population size. However, few have investigated the causal interaction between NTL and socio-economic development. Analyzing the causal relationships between NTL and socio-economic variables can be more valuable than traditional correlation approaches for understanding the drivers of city growth, as well as prioritizing long-term policy drafting and practical urban planning. For example, Hoffmann et al. [17] tested the causal relationship between Foreign Direct Investment (FDI) and pollution level, discovering a significant direction of causality from FDI to CO_2_ level in middle-income nations. Seto & Kaufmann [18] examined socioeconomic drivers of urban land use changes in the Pearl River Delta using high spatial resolution satellite data and demographic records from 1988 to 1996.

In this work, we extend the spatial coverage to 25 cities in pan-Pacific region using annual composites of NTL composite from 1992 to 2012. We first intercalibrate NTL data using a localized modelling approach to ensure temporal consistency and minimize any effect caused by saturated NTL pixels [19]. Second, we spatially delineate and track urbanization patterns using the Theil-Sen estimator for 25 urban environments across the pan-Pacific region. Then, we examine the causality of two common socio-economic variables (population and GDP) on NTL using panel Granger causality procedures. This approach allows a statistical verification of the possible drivers of urban development by examining the effect of exogenous macro-level socio-economic factors on physical city growth. This work demonstrates new ways of investigating relationships between NTL data and socio-economic development.

## Materials and methods

### Study area

We selected 25 urban environments across the pan-Pacific region, covering 12 countries across a broad spectrum of population size, economic, and ecological conditions. Urban environments located in less developed regions (e.g. Eastern and South-eastern Asia, and South America) were expected to have greater increasing anthropogenic activity level (i.e. increasing nighttime lights) compared to more developed areas (e.g. North America). Mega-cities such as Manila, Mexico City, and Tokyo were also included to represent urban changes in highly populated areas with contrasting economic conditions. Ecologically, urban environments such as Las Vegas offer a unique perspective on how cities develop in less suitable environmental conditions (e.g. arid environments). We also included urban environments such as Changsha and Nanchang, China, which are not often in the spotlight of urban studies but are of critical cultural and economic importance.

### Intercalibrate nighttime lights time series

Urban boundaries, particularly administrative boundaries, are often vaguely defined and highly dependent on local jurisdiction systems [20]. Rather than using an administrative boundary, a 60-km radius circular buffer was generated for each urban environment. Waterbodies were masked out from subsequent analysis using a previously generated water mask [21,22]. Annual average visible cloud-free nighttime lights composites (Version 4) were acquired from NOAA (http://ngdc.noaa.gov/eog/dmsp.html) covering 1992–2013. Images were formatted as Digital Numbers (DNs) ranging from 0 to 63, with a higher DN representing greater illumination or brightness of lights.

Due to the lack of an onboard calibration mechanism, robust intercalibration is a critical step to allow images from different years or sensors to be directly comparable. Recently Pandey et al. [23] quantitatively evaluated nine most commonly used intercalibration techniques using a Summed Normalized Difference Index (SNDI, Equation 1). Similar to Zhang et al. [24] and Elvidege et al. [25], we built a 3^rd^ degree polynomial model to calibrate each image to a reference year. A reference year is selected based on maximal DN values across the selected cites, an approach which has been used previously [25–29]. Rather than using one single model for all cities, we fit a polynomial model for each individual city to account for local NTL variations.

We evaluate our calibration results for each city using SNDI, which quantifies the level of convergence in NTL temporal series of a given city (2017). SNDI is the total of Normalized Difference Index (NDI, Equation 2) and assesses the absolute difference of total DN values (TDN, Equation 3) between two sensors in the same year. As suggested by Zhang et al. [24] and Pandey et al. [23], an effective intercalibration should yield a much lower SNDI than the raw images. Our intercalibration SNDI was then compared against raw data, Zhang et al. [24], and Elvidge et al [25].

$$SNDI=\sum_{i=1}^{11}{NDI}_{t}$$ (1)

$${NDI}_{t}=\frac{\left|{TDN}_{1t}-{TDN}_{2t}\right|}{{TDN}_{1t}+{TDN}_{2t}}$$ (2)

$$TDN=\sum_{i=1}^{n}{DN}_{i}$$ (3)

tϵ(1997,1998,1999,2000,2001,2002,2003,2004,2005,2006,2007)

1tand2trepresentNDIvaluesatagiventimetfrom2differentworkingsensors

A challenge associated with NTL data is pixel saturation, which can occur due to the limited radiometric range of NTL sensors. Recently Zhang et al. [30] incorporated a series of vegetation images to de-saturate NTL data on the assumption of an inverse relationship between vegetation abundance and NTL brightness. However, since our goal of this study is to investigate the casual relationship between NTL, GDP and population, the inclusion of another input variable (e.g. vegetation) complicates the process of interpreting statistical analysis. In addition, Zhang et al. [30] suggested a limited improvement of NTL variability for fast growing cities compared to more established legacy cities. The inverse relationship between NTL and vegetation may not hold for developing cities in this study. As a result, NTL images used in this study are calibrated but not alerted to accommodate potential saturation issues.

### Generate NTL temporal trend

As indicated by previous studies, a “lit” pixel does not necessarily coincide with human activities due to the potential “blooming” effect caused by diffused or scattered light from neighboring pixels [31]. We therefore used a DN of 12 as a threshold between lit and non-lit, or dimed, pixels [32]. We then generated NTL trends for all 25 urban environments over the 21 years. In order to capture any development in initially low-lit areas, NTL trends were generated for all non-water pixels, including the ones with a DN value below 12. A Mann-Kendall non-parametric test [33] was used to determine the significance of the monotonic trend in NTL. The TS estimator, which has been widely used with time series data [34,35] to describe temporal change in intensity, was applied to pixels identified by Mann-Kendall as statistically significant (p < 0.05). Those pixels were then used to calculate the trend slope values based on the median of pairwise data points from 1992 to 2013.

Based on the slope values, we then grouped the 25 cities into two classes. The first class contained cities that have experienced rapid NTL increase over the 21-year period. The second class represented cities with much lower or no slope trend in NTL, indicative of little urban growth over the time. We also examined the NTL with a predefined threshold (e.g. DN = 12) to determine in which year a given pixel exceeded the threshold value and its urban establishment passed the brightness threshold.

### Granger causality test

Although NTL has been extensively used as a proxy to anthropogenic activities, sophisticated and well-tested econometric tools have rarely been applied with the NTL time series. The most notable challenge is that econometric tools often require decadal or even centurial time series as input in order to capture the often weak relationship between two given economic variables [36]. NTL imagery collected by DMSP/OSL has a relatively short time span (i.e. 1992–2013) and therefore is often not well suited for econometric tools. Recent studies [37], however, have shown encouraging results for utilizing relatively short time series of data for causation testing through panel data that are a collection of entities (e.g. cities), where the variables are observed across time.

Statistically, the Granger causality test [38] describes the strength of association between two time series by testing whether or not the inclusion of one time series (x_t_) can improve the forecasting of future values in another time series (y_t_). If the addition of x_t_ significantly improves a model’s explanatory power in predicting y_t_, we could conclude that x_t_ “granger causes” y_t_.

The panel version of the Granger causality test combines individual short-time series data in the form of cross-sectional structures that increase the test efficiency and power by raising the number of observations and degrees of freedom [17]. In this work, a total of three panel data sets were generated for causality testing, namely, total DN (*T*_*DN*_), total population (T_*POP_Total*_), and total GDP (*T*_*GDP_Total*_), for each of the 25 cities (see data source in Appendix 1). As a result, each panel data set had a total of 25 cross-sections (N = 25 cities) and 22 temporal units (T = 21 years). Total DN was calculated as the sum of DN values of all lit pixels for each year to represent both the area and intensity of the NTL. We also examined the differences between cities that are rapidly developing (N = 13) versus those that are more established (N = 12).

Granger causality tests require all panel data to be stationary and co-integrated based on two panel unit root tests; the Levin & Lin [36], known hereafter as the LLC test and Im & Pesaran [39], known hereafter as IPS. The panel co-integration test of Johansen [40] was applied to examine co-integration among all pairs of temporal variables (see test results in Appendix 2).

The rejection of Granger causality tests H_0_ (p < 0.01) indicates a unidirectional causal relationship from one input variable to the other. We employed the panel Granger causality test proposed by Dumitrescu and Hurlin [41], hereafter DH, which respects the heterogeneity within relatively small panel data sets.

## Results

### Intercalibrating NTL time series

**Fig 1:**
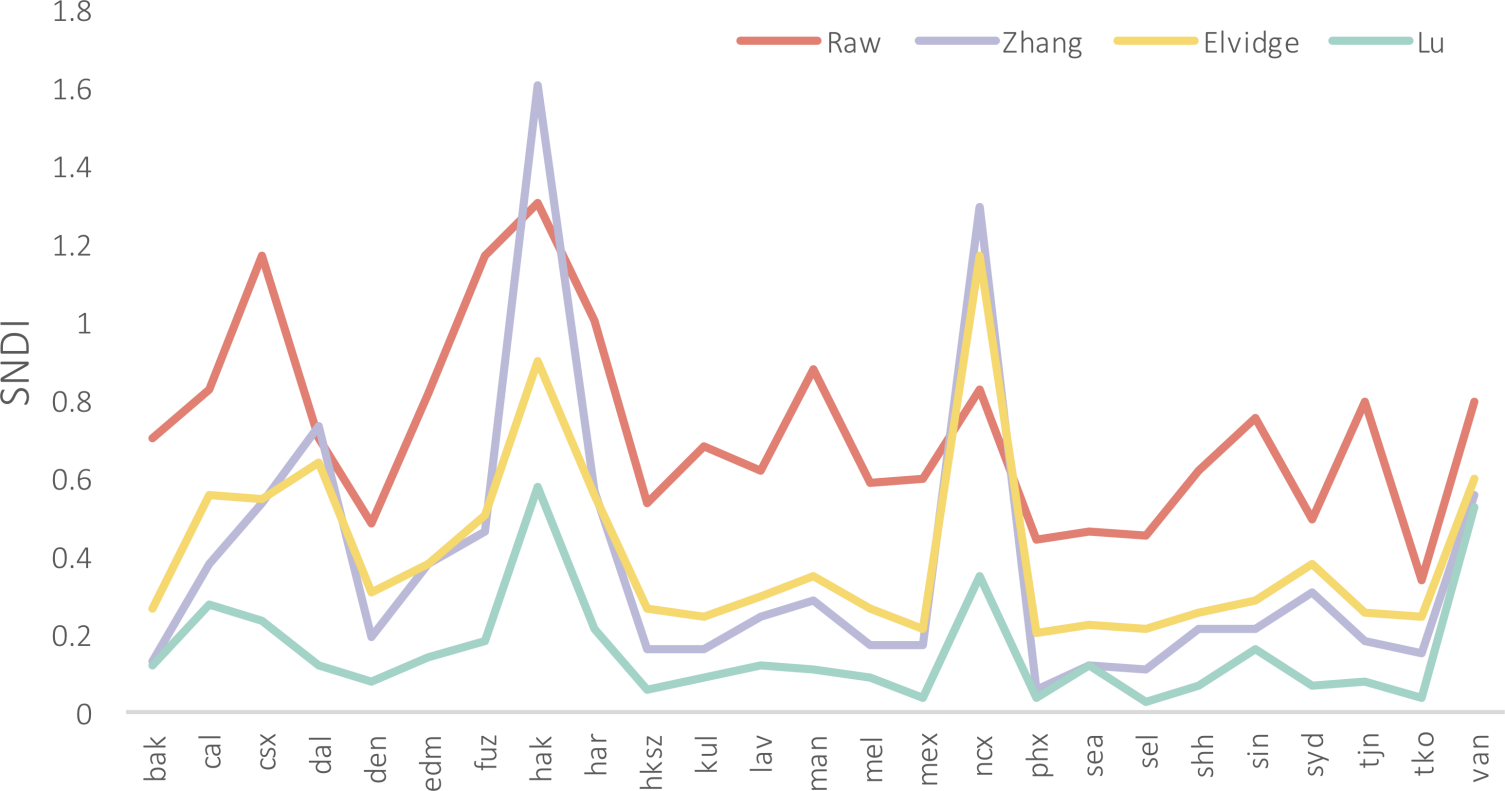
Sum of normalized difference index (SNDI) derived from raw images, Zhang et al., (2016), Elvidge et al., (2014), and Lu (this paper).

Overall, all calibration methods successfully reduced the systematic biases in the NTL images with a lower SNDI than for the raw data across most cities (Fig 1). Although Zhang et al. (2016) and Elvidge et al. (2014) yield lower SNDI at the global scale, our city level calibration shows a marginally better calibration result in terms of minimizing systematic biases. Haikou (HAK), Nanchang (NCX), and Vancouver (VAN) all have a relatively higher SNDI value compared to the other cities tested.

### Quantifying spatio-temporal changes

**Fig 2:**
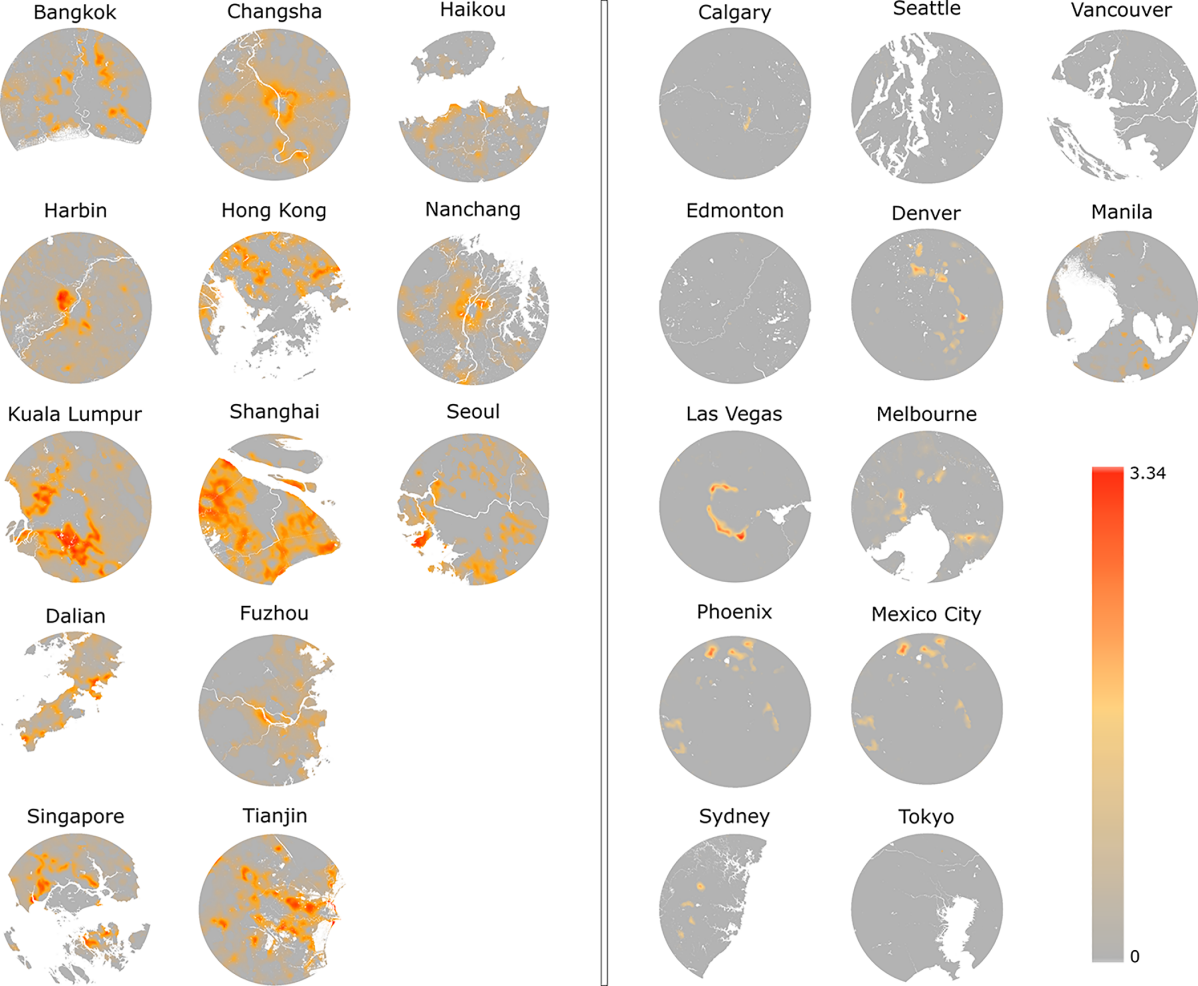
NTL change rate represented by Theil-Sen slope values showing the rate of change from 1992 to 2013. Water is colored as white. Cities were grouped based on their growth intensity. Left panel contains cities with fast and more dynamic urban growth, while the right panel includes cities with more stable and less development.

Large inter- and intra-city variations are apparent; for example, in Denver (DEN), steeper slopes are clustered in the north and east of the city while in Kuala Lumpur (KUL), intensive NTL changes are located in the south (Fig 2). The majority of pixels with rapid change are found in less developed cities (e.g. HAR) while more developed cities exhibit more stable NTL trends (e.g. CAL). Variations within a city also clearly highlight NTL change hotspots, such as the growth of surrounding satellite cities during the study period (e.g. BAK, SHH).

**Fig 3:**
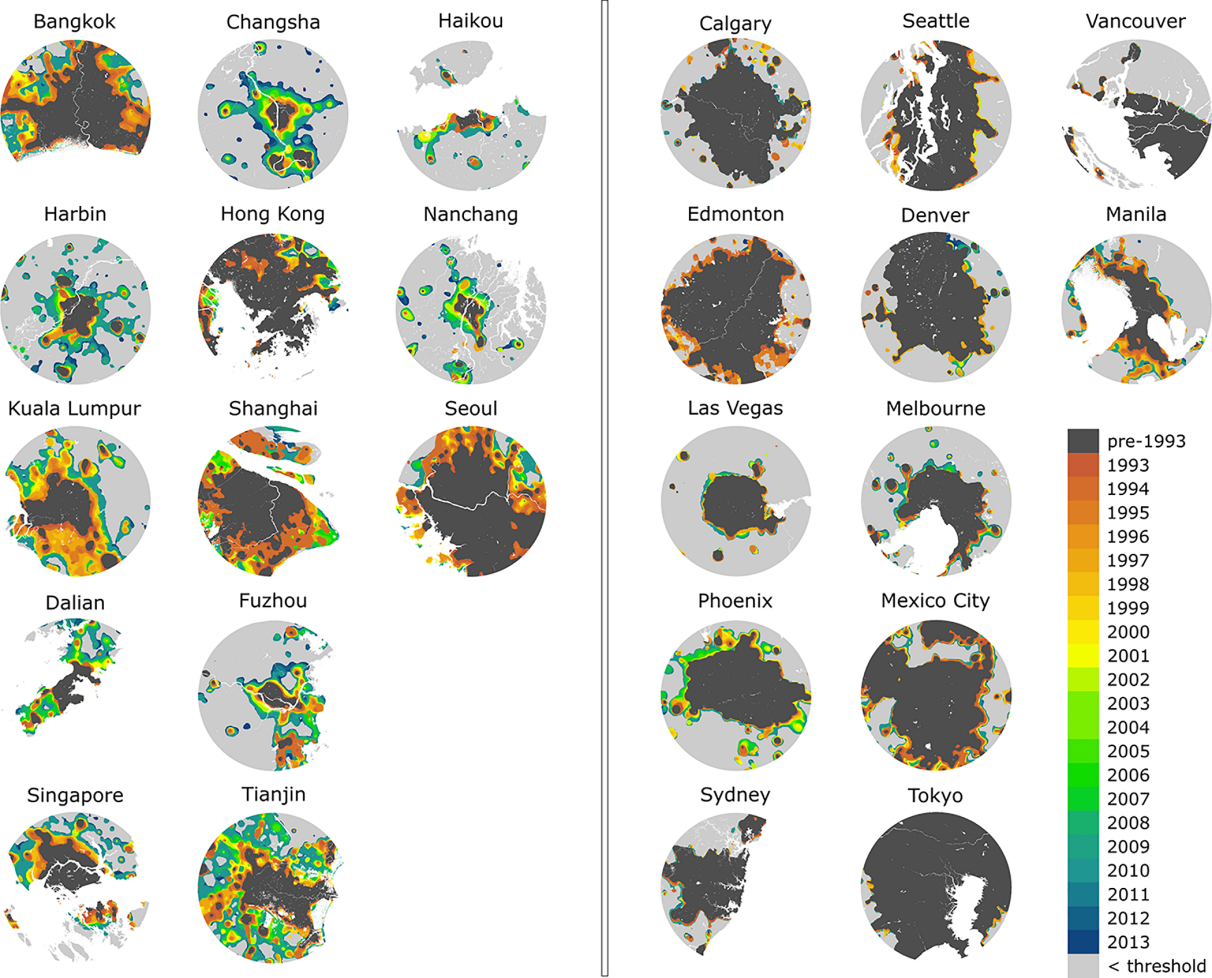
The year when a given pixel within each urban environment exceeded the pre-defined DN value. Dark grey pixels represent existing urban areas prior to 1992, while light grey indicates areas with no sufficient light sources in 2013.

Spatially, the recent urban development generally occurred on the outer rings of each urban area (Fig 3; e.g. FUZ and CSX). Timing of urban development was also variable with cities, such as Seoul and Kuala Lumpur, which were dominated by land cover changes in the early stage of the time series, while changes in Changsha and Dalian were relatively more recent.

**Fig 4:**
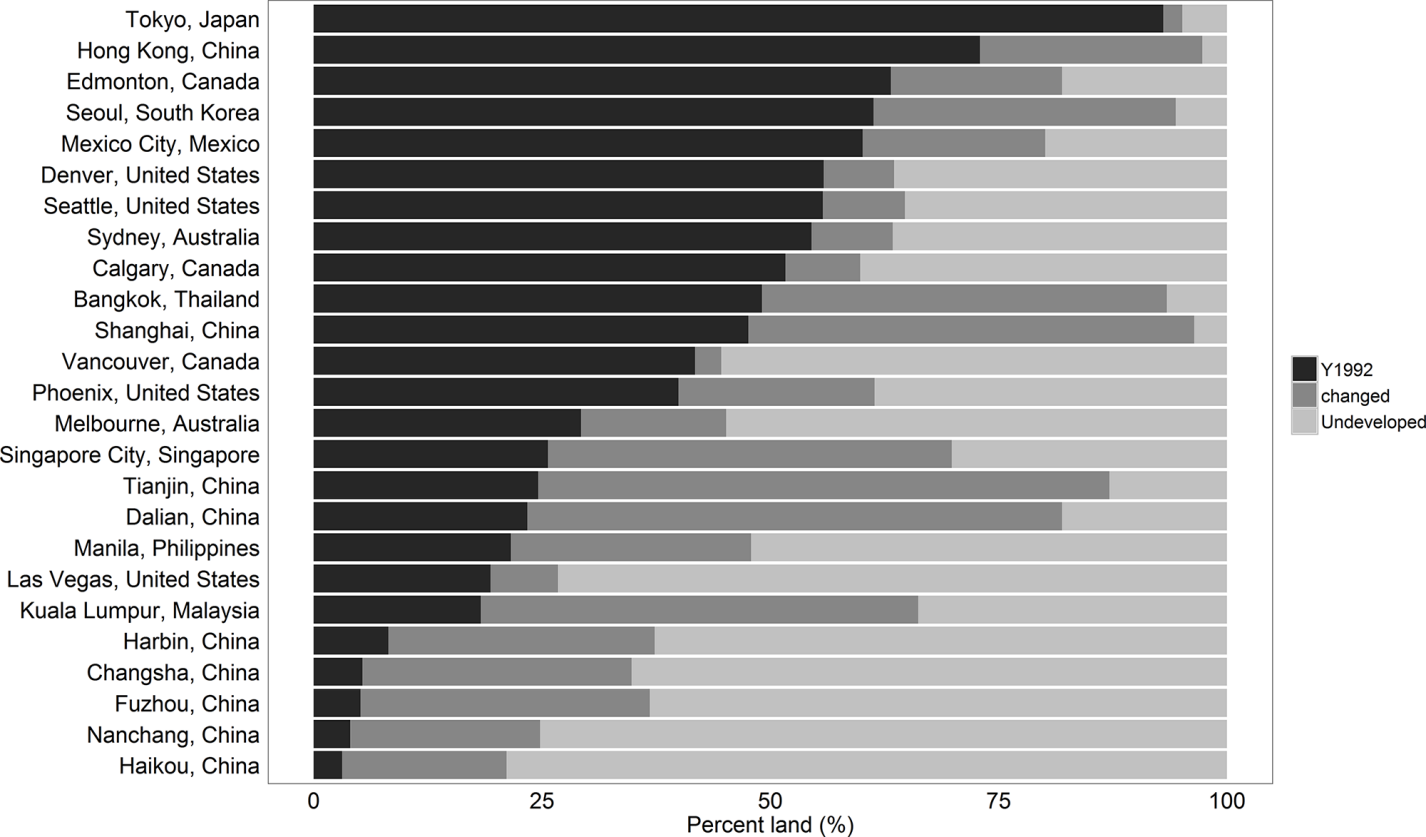
Urban land breakdown of changed, undeveloped, and existing urban areas, ranking the 25 cities from the highest proportion of lit pixels (i.e. TKO) to the least (i.e. HAK).

We observed a wide range of variation within and across all 25 cities in urban development (Fig 4). For example, Tokyo (TKO) and Shen Zhen—Hong Kong (HKSZ) have over 75% of land urbanized prior to 1992 while most cities in China have less than 10%. Cities such as Shanghai (SHH) and Tianjin (TJN) experienced substantial growth over the period studied with nearly 50% of their land crossing the pre-defined threshold value. A few cities, however, had less growth with approximately 75% of land remaining undeveloped.

### Granger causality test

**Fig 5:**
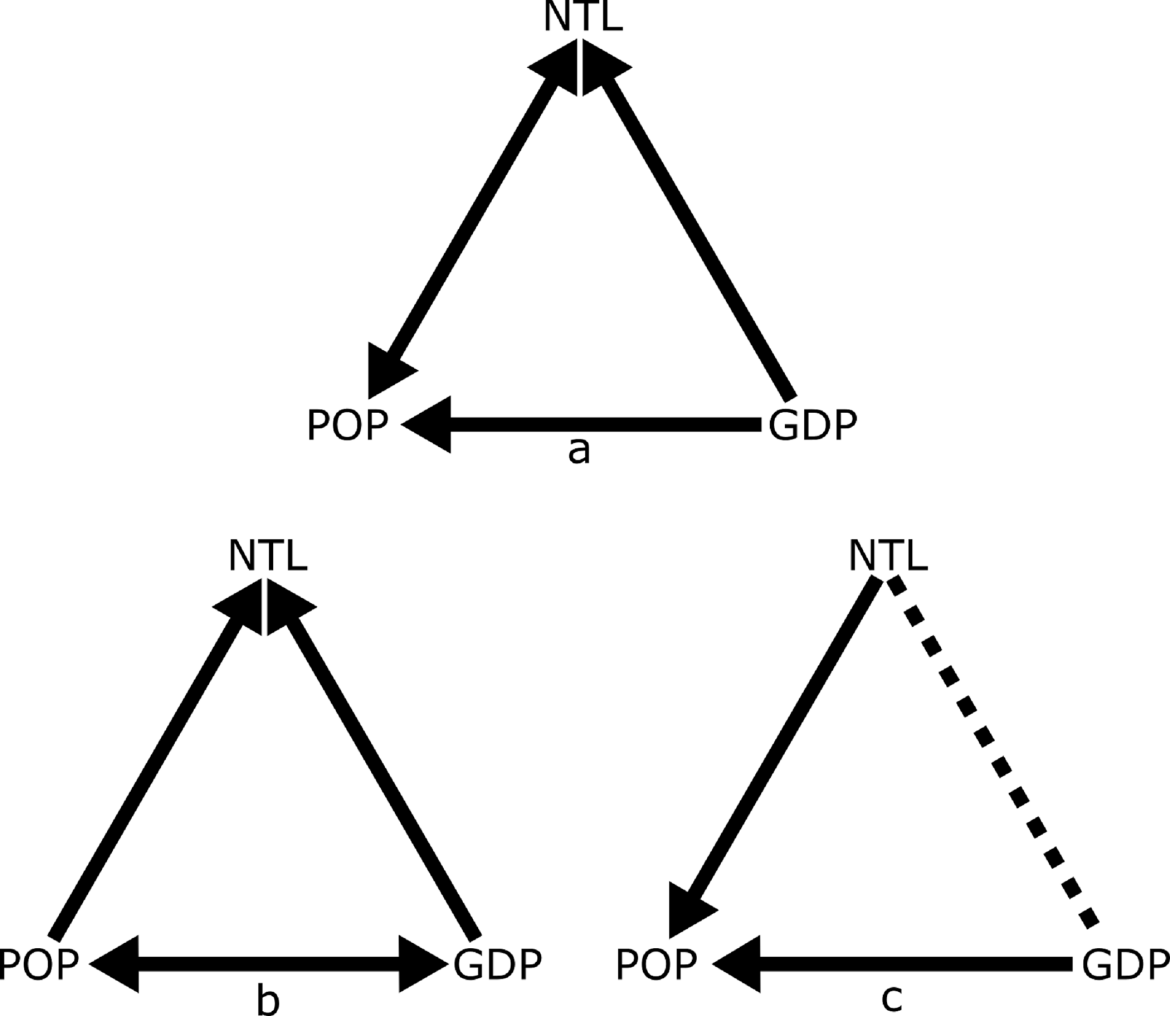
Causal interactions among NTL (nighttime lights), POP (population size), and GDP (Gross Domestic Product) of a) all cities, b) established cities, and c) dynamic cities. A solid line represents a statistically significant causal relationship, while a dotted line indicates no significant causality. An arrow head indicates the direction of causal relationship, and a double-headed arrow represents a bi-directional causal relationship.

The causality test results differ depending on the city analyzed (Fig 5). Expectedly, across all cities, both population and GDP play a major role in directing changes of NTL. Additionally GDP and NTL also “granger cause” changes in population (p < 0.01) (Fig 5A). This implies that the brightness of cities follows increases in both population and GDP equally and that neither population nor GDP alone is responsible for increasing the NTL. Among all cities, the test also unexpectedly suggests GDP and NTL “granger cause” population growth suggesting that population change is the outcome rather than the cause of urban development (Fig 5A).

After stratifying the cities by development stage we find contrasting and unexpected results. In the case of more established cities with few NTL changes over the analysis period, the causal relationship from NTL to population is no longer significant yet changes in population “granger cause” both GDP and NTL (Fig 5B). This suggests that in cities with relatively stable NTL, population and GDP are likely the key drivers of local economic and urban development, and not the other way around.

For fast changing and more dynamic cities there are only two significant casual relationships–growth in NTL or GDP leading to an increase in population. Unexpectedly, there is no significant causal association between GDP and NTL (dashed lines in Fig 5C). This suggests that in rapidly changing cities population increases are driven by brighter and more economically active urbanization.

## Discussion

### Intercalibration of nighttime time series

Our calibration method successfully minimizes the systematic biases at the city scale, enabling direct comparisons among images taken by different sensors (Fig 1). Pandey et al. [23] suggests that a global calibration (i.e. national level) could outperform regional models, however this does not appear to be the case in this study. We found that intercalibrated models at the city scale achieved relatively lower SNDI across all 25 cities than using calibration parameters from previous studies (Fig 1). One rationale is that the majority of the pixels used in the calibration process are brightly lit (i.e. pixels located in urban areas), which have a much higher contribution to the overall SNDI statistics than dimly lit pixels [23]. Future studies could focus on developing a more systematic procedure to select reference images and empirical models to accommodate for the need of individual studies at varying spatial scales. It is also noticeable that the calibration performance varies across cites (Fig 1). Images with larger portions of dimly lit pixels are more likely to suffer from less optimal calibration due to the existence of random noise and the skewed radiometric DN values. Island cities or cities surrounded by large green spaces may have a relatively less even distribution of DN values, which may explain our inconsistent calibration performance in Fig 1. Cities with higher SNDI values are either located in developing regions (e.g. Changsha and Haikou) or cities with higher cover of vegetation cover (e.g. Vancouver). Generally, those cities have fewer brightly lit pixels than cities such as Tokyo. Therefore, we conclude that a locally fitted intercalibration model will likely work better in areas dominated by high DN pixels. Future studies involving areas with a substantial amount of dark pixels may need a verified image histogram before selecting any intercalibration technique.

### Is a 60-km buffer enough?

It is unsurprising to see that cities with more dynamic and fast changing rates are located in Asia. According to United Nation’s review in 2001, on average, Asian cities are at least 50 years behind Europe and North America in terms of their urbanization level [42]. Mega-cities in Asia on the other hand show highly dominating and disproportional impact on regional and national economic development. Studies [43] have suggested that urban dwellers have an overall better living standard, such as education and consumption level, hence attracting a substantial amount of migrants from rural areas. Understanding the spatial pattern and the timing of urban development in these fast changing cities can offer valuable information on efficient land resources allocation, which can further reduce the per capita cost of infrastructure and basic services [44]. In contrast, urbanization tends to be less concentrated in more developed cities due to their advanced urban network [44]. As a result we noticed that while a 60-km radius buffer was sufficient for fast changing and more dynamic cities, it is clearly not large enough to capture recent urbanization activities in more developed cities (Figs 2–3). Other alternatives such as algorithmically derived urban extent have been used in previous literature, focusing on primarily tracking urban land cover and land use over time. This approach, however, still requires a fixed boundary to define where the city ends.

Although this study uses a ground distance of 60 km to delineate the urban boundary, other distance measuring approaches such as travel time ratio [45] or Manhattan distance [46] may affect the casualty tests. One limitation regarding the boundaries of selected cities is the spatial scale difference between remote sensing data and census record, which is often collected using administrative units. The scale inconsistency between these two data sets may alter the final results. Yet, since the census data used in this work represents the metropolitan area, which in general covers a relative larger area than normal administrative units, we believe our results are still valuable in decoupling the relationship between NTL and socio-economic development.

### Chicken or the egg: Causal relationship between NTL and socio-economic factors

A large number of econometric studies have reported inconsistent results when examining interactions between socio-economic and environmental variables. Mozumder and Marathe [47] summarized a number of studies and found mixed causal relationship results depending on the study location, types of variables, and duration of time series used. Knapp [48] tested the underlying interaction between population growth and global CO_2_, and concluded a weak long-term equilibrium but strong short-term relationships between population and CO_2_. Seto & Kaufmann [18] also employed panel causality procedures with remotely sensed images to estimate the economic drivers of land conversion in urban areas and concluded that investment in capital construction is driving urban land conversion. It has long been thought that population is the primary driver of urban growth while economic development is rather an outcome of urbanization. In this work, we found that population and GDP revealed contrasting effects on NTL trends between stable and more dynamic cities. Statistically, changes in NTL are significantly driven by both population and GDP growth in more established and slow changing cities. Previous work [49,50] has indicated that rather than growing population alone, it is the high consumption lifestyle, as well as economic and political decisions that lead to urban growth. Our results also show that in more developed cities it is in fact both the population and economic development that drives urban growth.

However, in fast changing, yet often less developed, cities the growth of NTL and GDP are driving population changes rather than the other way around. In those cities, a major source of population increase is through large in-migration from rural and neighboring areas, and involves densification and conversion of existing farm, forest, or barren land to urban land cover types [43]. Our results suggest that migration is more attracted to cities with promising economic conditions and undergoing fast urbanization paces.

Consistent and comparable measurement is key to evaluating sustainable urban development. Satellite derived images produce spatially explicit profiles of cities’ development. The integration of time series of satellite images and conventional census-based measurements reveals new ways of monitoring urban development, offering continuous and calibrated measurements of city brightness. Combined with temporally consistent variables, this work also offers valuable input toward local urban planning, such as resources allocation and public transportation networking [51]. Furthermore, given the nature of satellite-derived data, such information is also highly comparable for large-scale cross-city comparison studies.

### Future research

The new nighttime lights images from the Visible Infrared Imaging Radiometer Suite (VIIRS) Day/Night Band (DNB) extend the timeline of such research to 2017 with more spatially and radiometrically improved data. Using the proposed panel causality tests, researchers and urban planners can quantitatively evaluate effects of population and GDP on city brightness during more recent years. Information derived from this work not only offers insight into the performance of past urban development and local policy effectiveness, but also builds a strong foundation for guiding future sustainable development strategies. The extended time series of data also provides a better foundation for investigating temporal lag or delay between nighttime lights changes and real-time census derived variables [52–54]. The quantification of such delays could be an ideal candidate indicator to measure the effectiveness of local policy and development strategies.

Pervious studies [17,18] have also suggested the critical role of Foreign Direct Investment (FDI) in driving and reshaping urban development. However, FDI is not a common metric across all 25 cities. In developing regions, previous research indicated a bi-directional casual relationship between FDI and GDP [55]. Similar results were found in Li & Liu [56] where 84 countries were tested using panel data from 1970 to 1999. Although GDP is a common and justified metric representing a common and justified metric representing a city’s economic performance in this work, other metrics such as FDI could be further investigated to more comprehensively examine the relationship between city brightness and economic development.

## Conclusion

Contemporary cities are collectively more dynamic, multi-dimensional, and complex. Urbanization and its associated physical and socio-economic characteristics are interacting at a much faster pace and occurring much beyond local level. In this work, such characteristics are reflected by inter- and intra-city variations derived from the nighttime lights imagery for 25 cities in the pan-Pacific region. Panel causality tests were applied to statistically examine the long-run interaction between NTL and socio-economic variables. This work has the advantage of being intuitively appealing, as well as simple to reproduce and implement in practical urban planning and management. The derived information is critical for city planners to efficiently compare, visualize, and allocate resources, as well as policy-makers to quantitatively evaluate inter-and city development in a polycentric and highly connected global urban system.

## Supporting information

S1 Appendix Appendix 1 Census data sources summary for each city. Appendix 2 Granger causality test results.
(DOCX)
