# Supplementary material for: Bright lights, big city: Causal effects of population and GDP on urban brightness
Source: PLoS One. 2018 Jul 11;13(7):e0199545. doi: 10.1371/journal.pone.0199545 (PMC6040752; doi:10.1371/journal.pone.0199545)
Supplement: S1 Appendix — Appendix 1 Census data sources summary for each city. Appendix 2 Granger causality test results. (DOCX) [file pone.0199545.s001.docx]

**Appendix 1**

| **City** | **Source** |
| --- | --- |
| **Bangkok** | National Statistical Office of Thailand (<http://web.nso.go.th/en/stat.htm>) |
| **Calgary** | Statistics Canada Table 051-0056 (2001-2013) & Table 051-0014 (1992-2000) & Statistics Canada Table 381-5000 <http://www.statcan.gc.ca/pub/91-214-x/2009000/related-connexes-eng.htm>  <http://www.calgaryeconomicdevelopment.com/research-and-reports/demographics-lp/demographics/> |
| **Changsha** | National Bureau of Statistics of China <http://data.stats.gov.cn/english/easyquery.htm?cn=E0105> |
| **Dalian** | National Bureau of Statistics of China <http://data.stats.gov.cn/english/easyquery.htm?cn=E0105> |
| **Denver** | United States Census Bureau <https://www.census.gov/programs-surveys/popest/data/tables.html> U.S. Department of Commerce Bureau of Economic Analysis <https://bea.gov/iTable/index_regional.cfm> |
| **Edmonton** | Statistics Canada Table 051-0056 (2001-2013) & Table 051-0014 (1992-2000) & Statistics Canada Table 381-5000<http://www.statcan.gc.ca/pub/91-214-x/2009000/related-connexes-eng.htm> <http://www.edmonton.ca/business_economy/documents/InfraPlan/edmonton_cma_population(1).pdf> |
| **Fuzhou** | National Bureau of Statistics of China <http://data.stats.gov.cn/english/easyquery.htm?cn=E0105> |
| **Haikou** | National Bureau of Statistics of China <http://data.stats.gov.cn/english/easyquery.htm?cn=E0105> |
| **Harbin** | National Bureau of Statistics of China <http://data.stats.gov.cn/english/easyquery.htm?cn=E0105> |
| **Shenzhen** | National Bureau of Statistics of China <http://data.stats.gov.cn/english/easyquery.htm?cn=E0105> |
| **Kuala Lumpur** | Kuala Lumpur Population <http://worldpopulationreview.com/world-cities/kuala-lumpur-population/> |
| **Las Vegas** | United States Census Bureau <https://www.census.gov/programs-surveys/popest/data/tables.html>U.S. Department of Commerce Bureau of Economic Analysis <https://bea.gov/iTable/index_regional.cfm> |
| **Manila** | Philippine Statistics Authority <http://www.nap.psa.gov.ph/grdp/previousReleases.asp> <http://www.nap.psa.gov.ph/stattables/> |
| **Melbourne** | Australian Bureau of Statistics Community Profiles <http://www.abs.gov.au/websitedbs/D3310114.nsf/Home/2016%20Census%20Community%20Profiles>OECD Statistics <https://stats.oecd.org/Index.aspx?DataSetCode=CITIES> |
| **Mexico City** | World Urbanization Prospects <https://esa.un.org/unpd/wup/> |
| **Nanchang** | National Bureau of Statistics of China <http://data.stats.gov.cn/english/easyquery.htm?cn=E0105> |
| **Phoenix** | United States Census Bureau <https://www.census.gov/programs-surveys/popest/data/tables.html>U.S. Department of Commerce Bureau of Economic Analysis <https://bea.gov/iTable/index_regional.cfm> |
| **Seattle** | United States Census Bureau <https://www.census.gov/programs-surveys/popest/data/tables.html>U.S. Department of Commerce Bureau of Economic Analysis <https://bea.gov/iTable/index_regional.cfm> |
| **Seoul** | Seoul Statistical Yearbook <http://data.seoul.go.kr/dataService/boardList.do#none> |
| **Shanghai** | National Bureau of Statistics of China <http://data.stats.gov.cn/english/easyquery.htm?cn=E0105> |
| **Singapore City** | Department of Statistics Singapore <https://www.singstat.gov.sg/find-data/search-by-theme?theme=economy\|population&type=data> |
| **Sydney** | Australian Bureau of Statistics Community Profiles <http://www.abs.gov.au/websitedbs/D3310114.nsf/Home/2016%20Census%20Community%20Profiles>OECD Statistics <https://stats.oecd.org/Index.aspx?DataSetCode=CITIES> |
| **Tianjin** | National Bureau of Statistics of China <http://data.stats.gov.cn/english/easyquery.htm?cn=E0105> |
| **Tokyo** | Statistics Division, Bureau of General Affairs, TMG <http://www.stat.go.jp/english/data/jinsui/2.html>OECD Statistics <https://stats.oecd.org/Index.aspx?DataSetCode=CITIES> |
| **Vancouver** | Statistics Canada Table 051-0056 (2001-2013) & Table 051-0014 (1992-2000) & Statistics Canada Table 381-5000<http://www.statcan.gc.ca/pub/91-214-x/2009000/related-connexes-eng.htm> |

# Appendix 2

Panel unit root tests indicated that not all panel data sets were stationary in level. T_DN_ was stationary in level (p < 0.01) as indicated by both LLC and IPS procedures. However, the test results were mixed for other socio-economic panel time series. T_GDP_Total_ contained a panel unit root in level and achieved stationarity at 1^st^ difference. T_POP_Total_ was stationary in both levels and 1^st^ using IPS procedure, but contained panel unit roots in levels using LLC test. Given the mixed test results, we used only the 1^st^ difference panel data sets for subsequent analysis.

Unit root test result

|  | Level | 1^st^ difference | Level | 1^st^ difference |
| --- | --- | --- | --- | --- |
|  | LLC | LLC | IPS | IPS |
| T_DN_ | -2.94605 *** | -13.8317 *** | -4.26640 *** | -14.7862 *** |
| T_POP_Total_ | -0.15784 | -5.36286 *** | -2.65526 *** | -5.97486 *** |
| T_GDP_Total_ | 2.06186 | -8.51984 *** | -0.18361 | -8.16482 *** |
| 10% (*), 5% (**), 1% (***) | | | | |

The Johansen Fisher co-integration test indicated a significant presence of long-run relationships between T_DN_, T_POP_Total_, and T_GDP_Total_ . We rejected the null hypothesis – no long-run relationship, at the 1% significance level for all pairs of panel data sets. The existence of a longterm equilibrium allowed for testing Granger causality between nighttime lights and socio-economic changes.

Johansen Fisher Co-integration test result

| Time Series Pairs | H_0_: number of cointegration vectors | Fisher statistic  (trace test) | Fisher statistics  (max-eigen test) |
| --- | --- | --- | --- |
| T_DN_ ~ T_POP_Total_ | None | 140.6*** | 128.3*** |
|  | At most 1 | 55.27 | 55.27 |
| T_GDP_Total_ ~ T_POP_ Total_ | None | 144.6*** | 137.4*** |
|  | At most 1 | 73.44 | 73.44 |
| T_DN_ ~ T_GDP_Total_ | None | 202.0*** | 178.3*** |
|  | At most 1 | 66.83 | 66.83 |
| 10% level (*), 5% level (**), 1% level (***) | | | |
